# Differential effects of gender and age on dynamic subjective visual vertical

**DOI:** 10.1186/s42466-023-00266-4

**Published:** 2023-08-24

**Authors:** Johannes Gerb, Lena Padovan, Nicole Lehrer, Thomas Brandt, Marianne Dieterich

**Affiliations:** 1 German Center for Vertigo and Balance Disorders, Ludwig-Maximilians University, Marchioninistr. 15, 81377 Munich, Germany; 2 Department of Neurology, Ludwig-Maximilians University, Munich, Germany; 3 Graduate School of Systemic Neuroscience, Ludwig-Maximilians University, Munich, Germany; 4 Hertie Senior Professor for Clinical Neuroscience, Ludwig-Maximilians University, Munich, Germany; 5 Munich Cluster for Systems Neurology (SyNergy), Munich, Germany

**Keywords:** Static and dynamic subjective visual vertical, Age, Gender, Vestibular system

## Abstract

In a retrospective study, the data of direction-dependent deviations in *dynamic* subjective visual vertical (SVV) testing were analysed in 1811 dizzy patients (174 benign paroxysmal positional vertigo, 99 unilateral vestibulopathy, 67 bilateral vestibulopathy, 151 Menière’s disease, 375 vestibular migraine, 82 cerebellar disorder, 522 functional dizziness, 341 unclear diagnosis) and in 59 healthy controls. Major findings were (i) a significant gender difference with higher directional deviations in females over the entire range of age, (ii) a significant increase of directional deviations with increasing age for both genders and in all disease subgroups as well as in healthy controls, and (iii) a lack of significant difference of directional deviations between all tested diseases. Thus, the data allow no recommendation for performing additional angular deviation analysis in dynamic SVV testing as part of routine clinical management of dizzy patients. However, as shown in earlier longitudinal studies, it still appears reasonable that dynamic SVV in acute rather than chronic vestibular disorders may provide a useful instrument for the monitoring of acute unilateral vestibular tonus imbalances in the course of the disease.

Adjustments of subjective visual vertical (SVV) provide an easily performed and most sensitive clinical measure of acute unilateral peripheral or central vestibular lesions [1]. This is particularly helpful for topographic diagnosis of the side and/or the level of a circumscribed lesion from the labyrinths via the brainstem and thalamus to the parieto-insular vestibular cortex [2, 3]. Because of the crossings of the bilateral graviceptive vestibular pathways in the pontine and mesencephalic brainstem tegmentum, unilateral disorders of the labyrinth and the caudal brainstem cause ipsilateral SVV tilts, whereas upper brainstem damage causes contralateral SVV tilts [1–3]. Thus, SVV tilts indicate an acute vestibular tonus imbalance with spontaneous recovery by central compensation mostly within weeks [3]. In healthy subjects, the static SVV provides a reliable measure which remains stable up to high age [1]. Testing “static SVV” in the frontal roll plane is performed with different simple methods such as adjustments of a straight line in the center of a half-spherical dome with its inner surface covered by a random dot pattern (Fig. 1) [1, 3]. Rotation of the dome or pattern (clockwise, cw, or counterclockwise, ccw) about the line of sight induces a stimulus-dependent apparent body rotation (rollvection) with a limited body tilt, called “dynamic SVV” [4, 5]. Patients with pathological deviations of static SVV regularly exhibit a corresponding asymmetry of dynamic SVV with larger tilt angles according to the static tilt [1, 2]. Hence, the directional preponderance of dynamic SVV confirms the static data with a greater sensitivity along the course of an acute vestibular disorder, as could be shown in longitudinal studies [1, 5, 6]. The focus of the current study was on the differential effects of gender and age on the dynamic rather than the static SVV in patients with different chronic vestibular disorders compared to healthy controls.

**Fig. 1:**
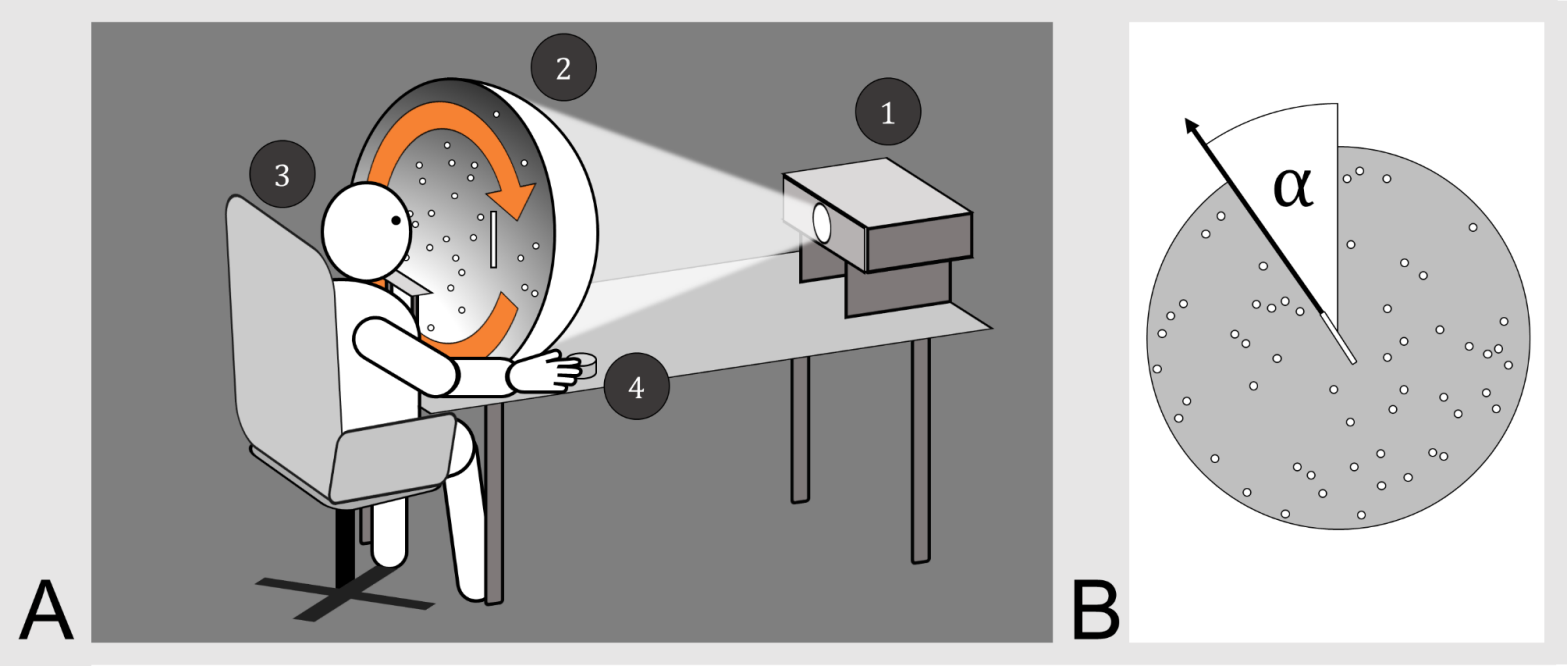
Schematics of dynamic SVV testing using the hemispheric dome method [1, 3]. **A**: a beamer (1) projects a dot pattern and a central line onto a spherical half-dome (2) which covers the entire visual field of the subject who is seated in front of the half-dome with head movement restricted by a chin rest (3). The central line is tilted in the roll plane at random intervals. The dots rotate in the roll plane for a minimum of 20 s in clockwise or counterclockwise direction (orange arrow); after 20 s, the subject is asked to orientate the central line from its offset position to the true verticality using a hand knob (4) while the dots continue to move. This measurement is repeated for multiple times in cw and ccw stimulus direction. **B**: The angular difference between the subject-submitted verticality and the true verticality is measured in 0.25° increments. An ipsidirectional stimulus-dependent angular tilt is physiological, whereas asymmetrical stimulus-dependent deviations are pathological. While dynamic SVV itself measures this difference between true verticality and the subject provided subjective verticality (defined as the mean of cw and ccw stimulus-dependent deviations), in this analysis we looked at the total angular deviation in clockwise and counterclockwise measurements by calculating the sum of mean *absolute* deviations

For many years testing dynamic SVV was part of routine clinical management of dizzy patients in the tertiary German Center for Vertigo and Balance Disorders, University of Munich, Germany. In the current retrospective study, we analysed the dynamic SVV data of a total of 1811 patients with different chronic or episodic peripheral or central vestibular pathologies (999 females, mean age 45.93°±16.12°, data collection period 2011–2023) who presented with normal static SVV, i.e., no current signs of acute vestibular tone imbalance. Diagnoses were benign paroxysmal positional vertigo (BPPV, n = 174), unilateral vestibulopathy (UVP, n = 99), bilateral vestibulopathy (BVP, n = 67), Menière’s disease (MD, n = 151), vestibular migraine (VM, n = 375), cerebellar disorder (n = 82) and functional dizziness (FD, n = 522). For control we included the data of 59 healthy volunteers (HC, mean age 35.13 ± 10.90 years); for the demographic analyses, we furthermore included 341 dizzy patients where no unequivocal final diagnosis was possible. All data were irreversibly anonymized and processed using Microsoft® Excel (Version 2022) and JASP (Version 0.17.1).

The major questions were the differential effects of age and gender and whether the sum of stimulus-dependent angular deviation in dynamic SVV is suitable to disclose and differentiate between patient groups with episodic vestibular syndromes (symptom-free interval) or chronic dizziness.

For simplification of data presentation, the individual mean stimulus-dependent absolute directional deviations of dynamic SVV were presented as the sum of cw and ccw testing. For further methods see Fig. 1 [1]. The interindividual variations were excessive ranging from a minimum of 1.25° to a maximum of 108.88°. Mean angular deviation showed a large variance in all subgroups (overall mean 32.05°±16.22°) and increased with age (Spearman’s rho 0.40 [95% confidence interval 0.36, 0.44], p < 0.001) (Fig. 2). Age-corrected ANCOVA-testing showed no significant difference between the different subgroups (mean total deviation: BPPV 35.10°±17.21°; UVP 32.88°±14.56°; BVP 36.13°± 15.85°; MD 34.64°±16.22°; VM 29.83°±15.42°; cerebellar disorders 37.83° ±16.43°; FD 29.78° ±15.33°; HC 22.10°± 12.05°). Figure 2 further exhibits a significant gender difference with larger deviations in females (mean difference male/female − 8.82°, p(Bonferroni) < 0.001, Cohen’s d -0.61) over the entire range of age and for all disease subgroups (not depicted). After correction for age and total deviation, no significant sex or disease differences for participant precision were found (defined as in-measurement standard deviation over all individually provided trials, ANCOVA with 1000 bootstraps and Bonferroni correction: mean difference male/female − 0.08° (95% CI -0.42°, + 0.48 °, pBonf 0.69).

**Fig. 2:**
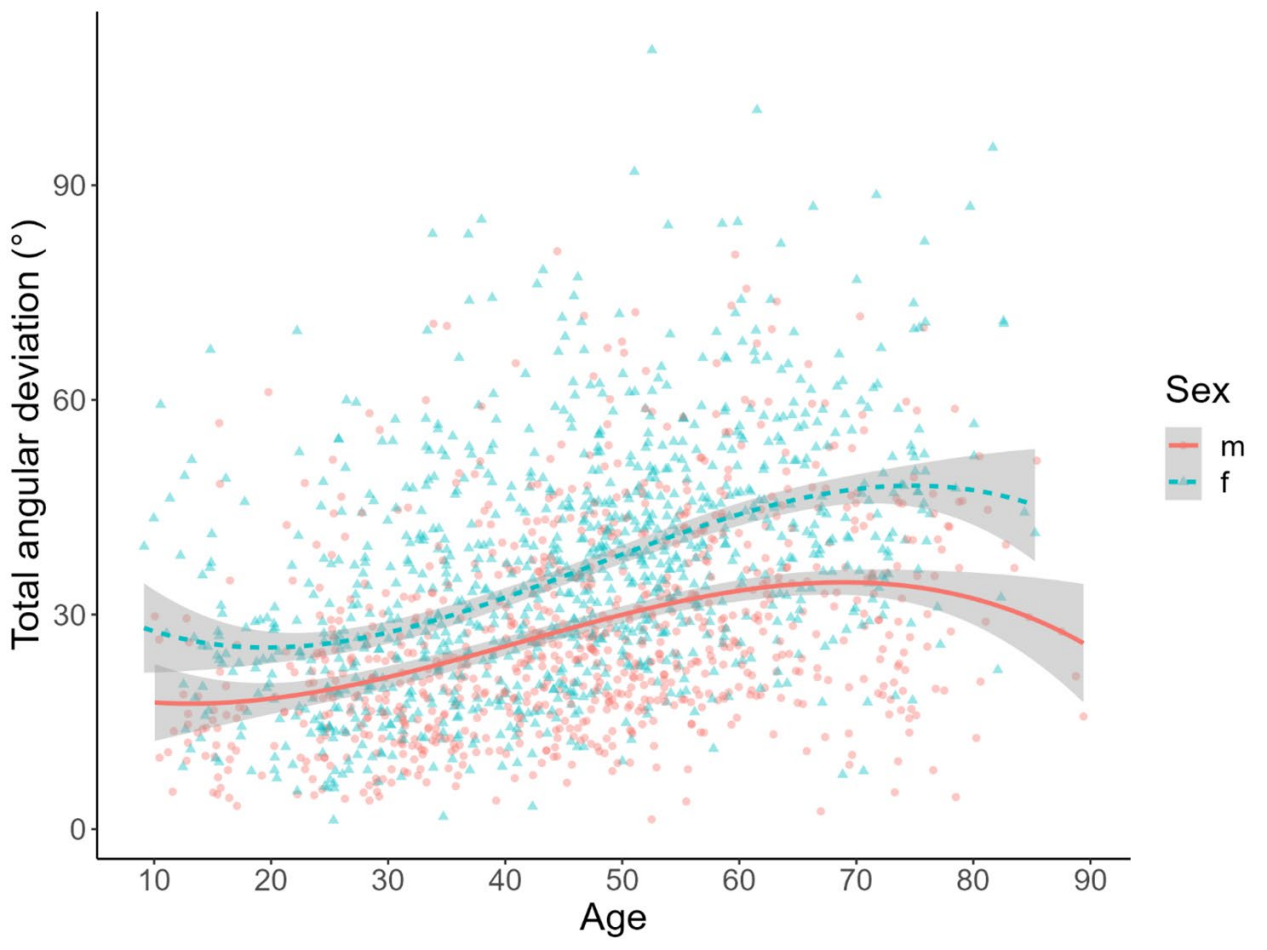
Plot of total angular deviation in dynamic SVV testing (sum of mean absolute clockwise values and mean absolute counterclockwise values) from 1870 participants (59 healthy subjects, petrol triangles: female participants, red circles: male participants, grey area: 95% confidence interval for gender-dependent mean total angular deviation). Over all participants, a large variance in the amount of stimulus-dependent angular tilt can be observed: on average, male participants (red line) are less affected than females (petrol line). Over the lifespan, an increase of susceptibility towards the visual circular vection stimulus in dynamic SVV was found

Earlier studies on smaller cohorts (n < 100) have already observed similar effects of gender [7] and age [1, 8] on dynamic SVV absolute tilt deviations and provided some evidence for a higher sensitivity of dynamic versus static SVV in the acute and subacute phases of unilateral vestibular lesions [5, 6]. The perception of subjective verticality is a basic precondition for spatial orientation and many studies have shown gender differences with females performing less precisely as compared to males [for review see: [9]. The latter authors differentiated between large- and small-scale spatial ability which they explained by gender specific emotional processes in the parahippocampal gyrus and the adoption of egocentric spatial strategies. Another study found increased dynamic SVV deviations in older adults with frequent falls which were interpreted as an increased visual dependence [10]. In contrast to an increased sensitivity to visual roll motion, as measured by dynamic SVV, in 66 male and female patients with VM [11] our retrospective evaluation of 375 VM patients did not confirm this finding.

In conclusion, our retrospective data of 1811 patients with episodic or chronic dizziness/vertigo does not allow the recommendation of stimulus-dependent roll-deviation analysis in dynamic SVV measures for the differential diagnosis of vestibular disorders solely because of the huge interindividual variations in clinical routine testing. The dynamic SVV itself (i.e., the analysis of the provided subjective vertical) may be helpful for monitoring the course of an acute unilateral vestibular loss based on several reports that the asymmetry of dynamic SVV persists longer than that of the static SVV [5, 6] and can be useful when confirming borderline pathological findings in static SVV measurement.

## Data Availability

The data that support the findings of this study are not publicly available due to patient and participant privacy, but anonymized datasets are available on reasonable request from the corresponding author [JG].

## Declarations

The study was performed in accordance with the ethical standards laid down in the 1964 Declaration of Helsinki and its later amendments.

## References

[1] Dieterich M, Brandt T (1993). Ocular torsion and tilt of subjective visual vertical are sensitive brainstem signs. Annals of Neurology.

[2] Brandt T, Dieterich M (1994). Vestibular syndromes in the roll plane: Topographic diagnosis from brainstem to cortex. Annals of Neurology.

[3] Dieterich M, Brandt T (2019). Perception of verticality and vestibular disorders of balance and falls. Frontiers in Neurology.

[4] Dichgans J, Held R, Young LR, Brandt T (1972). Moving visual scenes influence the apparent direction of gravity. Science.

[5] Faralli M, Ricci G, Molini E (2007). Determining subjective visual vertical: Dynamic versus static testing. Otology & Neurotology.

[6] Lopez C, Lacour M, Ei Ahmadi A, Magnan J, Borel L (2007). Changes of visual vertical perception: A long-term sign of unilateral and bilateral vestibular loss. Neuropsychologia.

[7] Goto F, Saito A, Araki Y, Kunihiro T (2003). Gender difference in roll vection. Equilibrium Res.

[8] Kobayashi H, Hayashi Y, Higashino K (2002). Dynamic and static subjective visual vertical with aging. Auris, Nasus, Larynx.

[9] Yuan L, Kong F, Luo Y, Zeng S, Lan J, You X (2019). Gender differences in large-scale and small-scale spatial ability: A systematic review based on behavioral and neuroimaging research. Frontiers in Behavioral Neuroscience.

[10] Totiliene M, Uloza V, Lesauskaite V (2021). Impaired subjective visual vertical and increased visual dependence in older adults with falls. Frontiers in Aging Neuroscience.

[11] Ashish G, Augustine AM, Tyagi AK, Lepcha A, Balraj A (2017). Subjective visual vertical and horizontal in vestibular migraine. The Journal of International Advanced Otology.

